# Visual psychological performance of different flattened bamboo boards based on semantic differentiation method and event-related potential

**DOI:** 10.3389/fpsyg.2026.1844749

**Published:** 2026-08-12

**Authors:** Caiping Lian, Xinlan Ruan, Lisheng Chen, Tong Tang, Meiling Chen, Xuehua Wang, Hong Chen, Shuqing Zhang, Zhihui Wu

**Affiliations:** 1 School of Design, Fujian University of Technology, Fujian, China; 2 Nanjing Forestry University, Nanjing, China; 3 School of Materials Science and Engineering, Guizhou Minzu University, Guiyang, China; 4 School of Art & Design, Qilu University of Technology (Shandong Academy of Sciences), Jinan, China; 5 College of Furnishings and Industrial Design, Nanjing Forestry University, Beijing, China; 6 International Center for Bamboo and Rattan, Beijing, China

**Keywords:** cognitive load, event-related potential, flattened bamboo boards, gender differences, material perception, semantic differential method, visual psychological perception

## Abstract

Flattened bamboo boards are increasingly used in interior environments; however, their influences on users’ perceptual and cognitive responses remain insufficiently understood. Using a sample of 25 participants, this study investigated the visual psychological impressions and neurocognitive processing associated with three types of flattened bamboo boards by integrating Semantic Differential (SD) evaluations with event-related potential (ERP) measurements. ERP analyses focused on the P300 component (300–500 ms) across frontal, central, parietal, and occipital regions. The results revealed clear perceptual and neural distinctions among the board types. The inner board consistently elicited the most positive perceptual impressions, being associated with naturalness, warmth, comfort, and delicacy, and was processed with the lowest cognitive load. In contrast, the outer board tended to be perceived as more artificial and decorative and evoked the highest P300 amplitude and longest latency, indicating greater cognitive effort and evaluative complexity. The bamboo outer layer board exhibited a more neutral and heterogeneous perceptual profile, with balanced evaluations of naturalness versus artificiality and exquisiteness versus roughness, while being more frequently associated with coolness and showing greater inter-individual variability in subjective evaluations. ERP analyses further demonstrated significant differences in P300 amplitude and latency across board types. While parietal P300 amplitudes differed significantly (*F* = 5.226, *p* = 0.049), latency differences were more pronounced in the central, parietal, and occipital regions (all *p* < 0.01). Gender-related differences were observed in both subjective ratings and neural responses, with male participants showing higher cognitive engagement during visual evaluation. Overall, these findings demonstrate that variations in bamboo surface appearance can systematically influence affective appraisal and cognitive load, providing empirical support for evidence-based material selection and interior design.

## 1 Introduction

Flattened bamboo boards are produced by softening bamboo culms at high temperatures, unfolding them, and shaping them under pressure. These boards offer several advantages, including a simple manufacturing process, high material utilization, large dimensions, adhesive-free composition, excellent dimensional stability, and superior mechanical properties (Fang et al., 2018; Yuan et al., 2021). During high-temperature softening, pyrolysis of chemical components within the cell walls causes the color of the flattened bamboo to deepen (Yuan et al., 2021), and coloration is an important quality attribute that influences consumer preference and the added value of bamboo products (Li et al., 2020). Owing to the two primary processing methods—removing or preserving the bamboo green and yellow layers—flattened bamboo boards exhibit a broad range of surface colors (Lou et al., 2022). Previous studies have shown that surface attributes such as color, texture, and gloss are key factors shaping visual appearance and eliciting psychological responses in humans (Liu, 2008). As flattened bamboo boards are increasingly used in residential environments, becoming part of occupants’ living surroundings, their visual presentation is expected to influence physiological and psychological states (Song, 2011; Li et al., 2018). Therefore, it is essential to investigate how different types of flattened bamboo boards give rise to holistic visual impressions and rational cognitive evaluations through perceptual and psychological responses.

Beyond material applications, environmental psychology research consistently demonstrates that visual properties of materials can influence affective appraisal, perceptual fluency, attentional engagement, and cognitive load (Reber et al., 2004; Zhou et al., 2025). Natural materials and biophilic elements often promote positive emotional states, whereas visually complex or low-fluency surfaces may demand greater cognitive resources and elicit evaluative ambiguity (Yin et al., 2018). Color attributes—such as warm versus cool tones—further modulate emotional valence and environmental comfort (Güneş & Olguntürk, 2020), while surface gloss and texture influence depth perception and scene interpretation (Anderson, 2011). Despite the relevance of these factors, empirical research linking bamboo surface microstructure, visual appearance, and cognitive processing mechanisms remains limited. This gap restricts our understanding of how bamboo materials shape user experience and environmental quality.

In response to this need, the present study focuses on three types of flattened bamboo boards and aims to determine how variations in surface attributes (e.g., coloration, texture, and perceived fineness) influence visual psychological evaluations and cognitive processing. Furthermore, we attempt to elucidate the underlying associations between visual perception and neural responses. Based on previous literature, we hypothesized that different surface characteristics would elicit distinct affective and evaluative responses; boards with warmer coloration and finer texture would evoke more positive impressions; and darker or more visually complex surfaces would demand greater cognitive resources. The overall research design and analysis were developed around these objectives and hypotheses.

Currently, the Semantic Differential (SD) method is widely used for subjective evaluation of material surface perceptions (Liu et al., 1995; Yu et al., 2004; Fujisaki et al., 2015). Visually perceived information is processed in the brain, influencing sensory judgments and decision-making. To enhance precision, some studies have integrated the SD method with Event-Related Potentials (ERPs) to assess individuals’ preferences for different objects (Zhang et al., 2021). However, research on the surface visual properties of materials remains limited.

ERPs are brain electrical responses recorded via Electroencephalography (EEG) that reflect sensory and cognitive processes triggered by stimuli such as visual patterns, textures, and material surfaces (Luck & Kappenman, 2011). The P300 component—emerging approximately 300 ms post-stimulus—is particularly sensitive to attention, stimulus evaluation, and decision-making, making it one of the most informative indicators of cognitive appraisal (Wang, 2016). Given its sensitivity to perceptual and evaluative demands, P300 provides a powerful measure for understanding how different material surfaces modulate cognitive processing.

Considering these strengths, this study integrates the SD method with ERP measurements to investigate the visual psychological perception and cognitive evaluation of different flattened bamboo boards. By linking material appearance with psychological and neural responses, this research seeks to advance theoretical understanding of material-based environmental perception and provide evidence-based insights for the application of bamboo boards in interior design and environmental esthetics.

## 2 Materials and methods

### 2.1 Materials

The flattened bamboo boards used in this study were sourced from three-year-old Moso bamboo (*Phyllostachys edulis*) harvested in Nanping, Fujian Province. They were prepared by Fujian Longzhu Group Co., Ltd. (Nanping, China) using a non-notched, high-temperature softening and flattening process with a softening temperature of 175–180 °C. The samples were classified into three categories:

1. Bamboo outer layer board – retains both the bamboo green and yellow layers.
2. Outer board – bamboo green surface removed (1 mm thick).
3. Inner board – bamboo yellow surface removed (1 mm thick).

**Figure 1:**
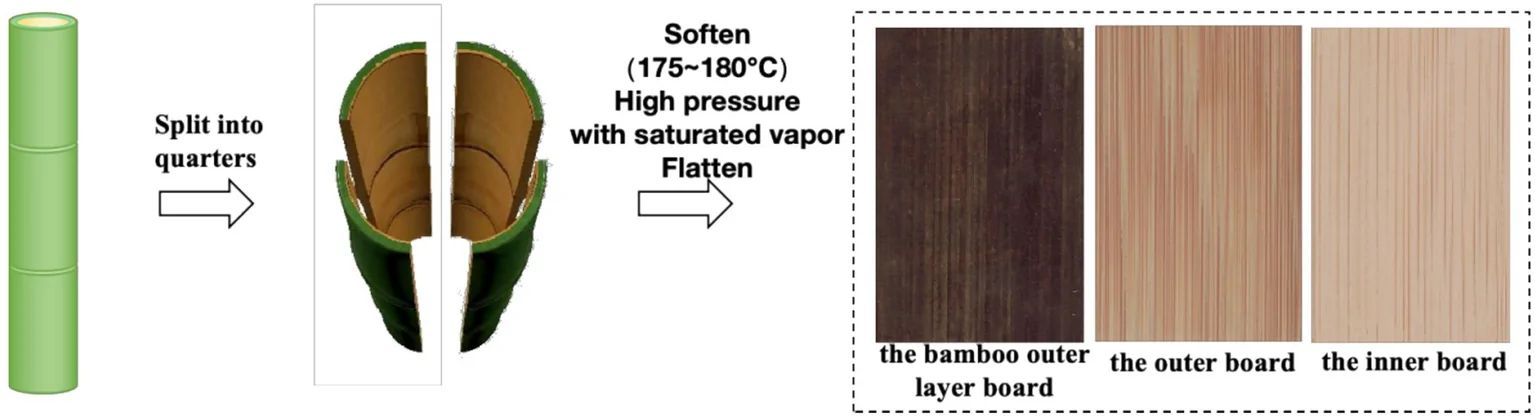
Schematic image of the sample preparation process and flattened bamboo board samples.

Each sample measured 60 mm × 40 mm × 5 mm (length × width × thickness), with a moisture content of approximately 8% (Figure 1). The specific production process and illustration regarding these were mentioned in our previous study (Lian et al., 2022).

### 2.2 Participants

The study recruited undergraduate students with relevant academic backgrounds, all of whom were physically healthy, had no history of mental illness, and possessed normal or corrected-to-normal vision. The participant group consisted of 25 young individuals with an average age of 23 ± 2 years (Standard deviation = 1.2), including 15 females and 10 males. The same group of participants completed both the Semantic Differential (SD) evaluation and the ERP experiment. The SD assessment was incorporated into the ERP experimental procedure to provide perceptual context for interpreting the neurophysiological responses.

Prior to the experiment, all participants were recruited voluntarily through an open announcement and were informed that participation was entirely optional and unrelated to any course requirements or academic evaluation. All participants received detailed instructions and signed written informed consent forms prior to data collection. The study received prior approval from the Science and Technology Ethics Committee of Fujian University of Technology (Approval No.: FJUT-2024004), and all experimental procedures were conducted in accordance with the committee’s ethical guidelines and relevant institutional regulations.

### 2.3 Semantic differential method

The Semantic Differential (SD) method was employed to evaluate participants’ psychological perceptions of the flattened bamboo boards. Following the “Three Dimensions” theory and the principle of bipolarity, an initial pool of 45 bipolar adjective pairs related to bamboo products was compiled from previous studies on the psychological perception of wood and bamboo materials (Song, 2011; Li et al., 2018). Participants were first asked to select the adjective pairs that they considered most appropriate for describing the visual characteristics of flattened bamboo boards, including color, texture, and gloss. Based on the selection frequencies, the 30 most frequently chosen adjective pairs were retained.

These 30 adjective pairs were subsequently classified into three dimensions—evaluative, sensory, and affective. Within each dimension, the adjective pairs were ranked according to their selection frequencies, and the two most representative bipolar adjective pairs were selected. Consequently, a final set of six bipolar adjective pairs was obtained and used to construct the semantic differential scale (Table 1). This procedure combined literature-based descriptor selection, participant screening, and dimensional classification to ensure that the selected semantic descriptors were representative of the visual perception characteristics of flattened bamboo boards.

**Table 1:** The classification results of semantic differential bipolar adjectives for flattened bamboo boards.

Evaluation	Sense	Emotion
Natural - Artificial	Smooth - Coarse	Comfortable - Afflictive
Practical - Decorative	Warm - Cool	Exquisite-Rough

**Figure 2:**
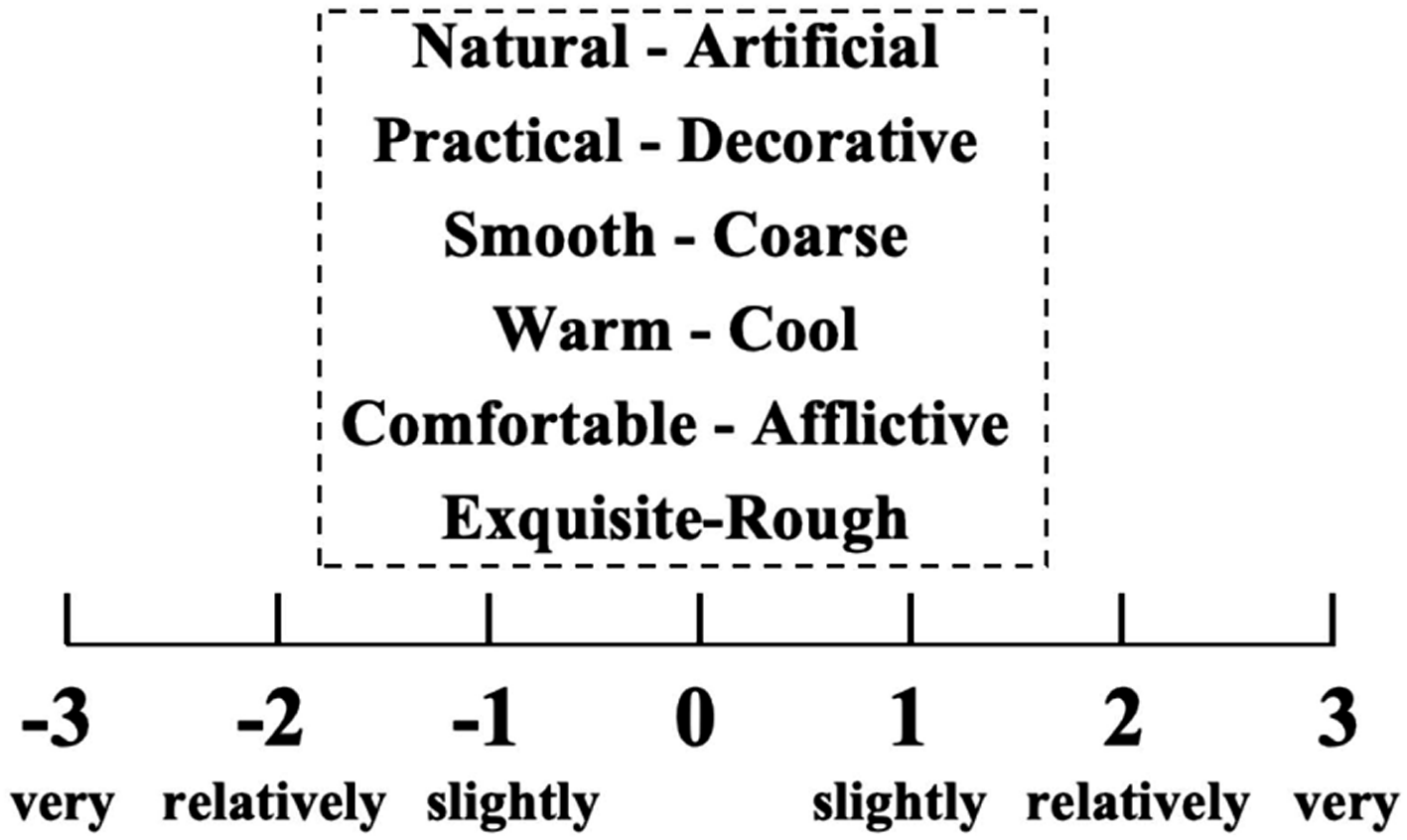
Semantic differential rating scale.

Each dimension comprised two pairs of bipolar adjectives, rated on a seven-point scale ranging from −3 to +3, with one-unit increments (Figure 2). To prevent directional response bias, the left–right positions of each adjective were randomized across trials. The experiment was conducted in an ergonomics laboratory, where participants were seated in a quiet environment to minimize external interference. Participants subjectively evaluated the samples based on their perceptual experience following the scale in Figure 2.

### 2.4 Event-related potentials

#### 2.4.1 Scheme design

To ensure consistency in visual stimulation, five images were prepared for each of the three types of flattened bamboo boards, producing a total of 15 images. Each image was paired with six bipolar adjective pairs, resulting in 90 trials (3 board types × 5 images × 6 adjective pairs). All images were standardized to 1920 × 1080 pixels and adjusted to accurately represent their real visual appearance.

In each trial, the image of the bamboo board was presented on the left side of the screen, while the corresponding adjective pair appeared on the right. Participants were instructed to choose the adjective that best matched their immediate visual impression by pressing the “F” key (left adjective) or the “J” key (right adjective). The stimulus display was self-paced, meaning that the image and word pair remained on the screen until a response was made. Once a response was registered, a blank screen was shown for 500 ms, after which the next trial was presented. The entire trial sequence was randomized for each participant using E-Prime 2.0.

**Figure 3:**
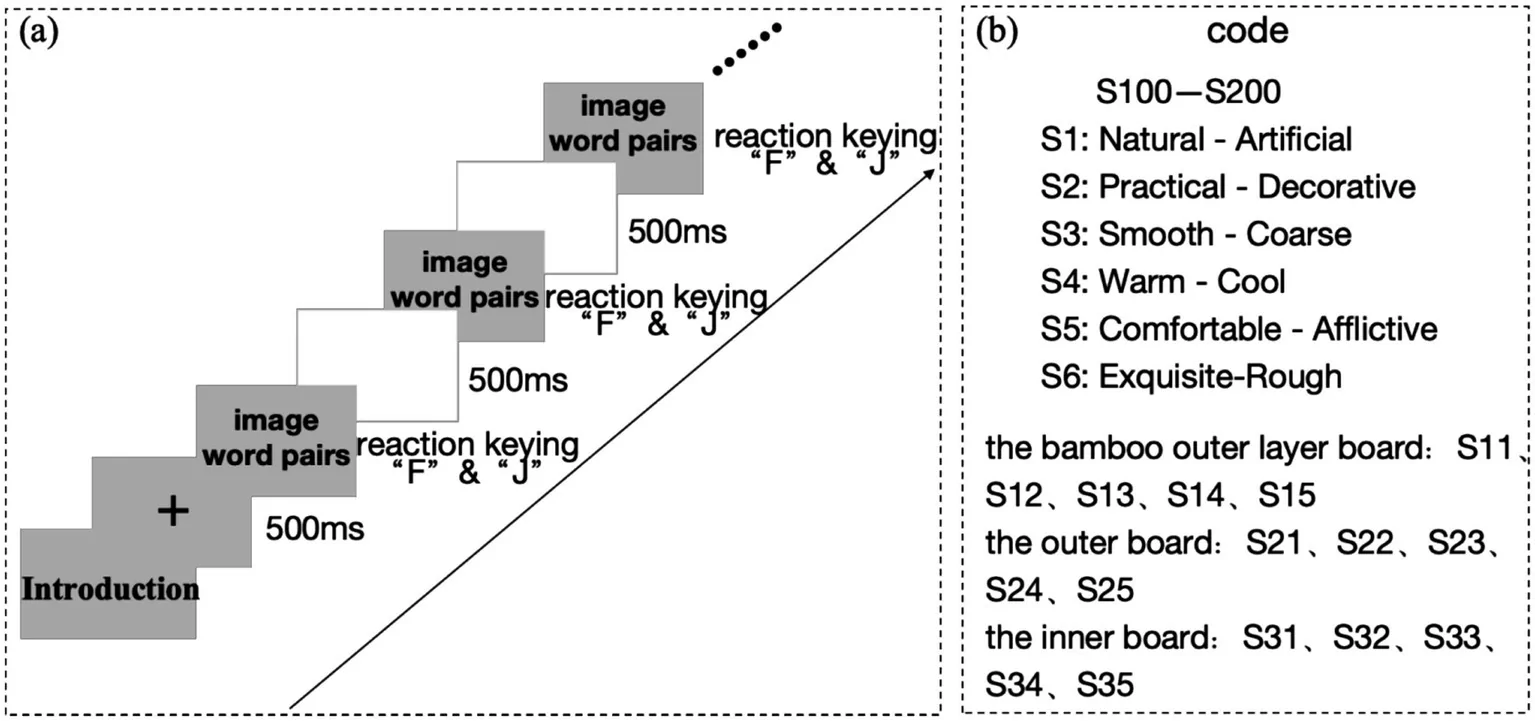
Experimental procedure. **(a)** Flow chart of the experiment; **(b)** The mark numbers corresponding to images and word pairs.

The overall procedure is illustrated in Figure 3a, and the corresponding E-Prime event markers are shown in Figure 3b. Each participant completed all 90 trials at their own pace, typically within 30 min, and the entire session—including EEG preparation—lasted approximately 1 h.

#### 2.4.2 Data acquisition and processing analysis

EEG signals were recorded using a 32-channel NeurOne system (Mega, Finland) with electrodes placed according to the extended 10–20 (10–10) electrode placement system, which is suitable for 32-channel configurations. Ag/AgCl electrodes were used, and all electrode impedances were maintained below 5 kΩ. The electrode layout included Fp1, Fp2, F3, Fz, F4, F7, F8, FT9, FC5, FC1, FCz, FC2, FC6, FT10, T7, C3, Cz, C4, T8, CP5, CP1, CP2, CP6, P7, P3, Pz, P4, P8, O1, O2, VEOG, and HEOG. Data were sampled at 500 Hz and referenced to the averaged bilateral mastoids (FT9/FT10).

EEG preprocessing was performed using Analyzer 2.1. Raw EEG data first underwent ocular artifact correction using EOG regression, followed by automatic artifact rejection based on an amplitude threshold of ±100 μV and gradient/low-activity criteria. Additional artifacts were removed through manual inspection, ensuring high data quality. The signals were then bandpass filtered between 0.1 and 30 Hz, and baseline correction was applied using the −200 to 0 ms pre-stimulus interval. Data were subsequently segmented according to stimulus markers and averaged to obtain ERP waveforms.

Each participant completed 90 trials (3 board types × 5 images × 6 adjective pairs). After artifact rejection, more than 85% of the trials were retained, providing sufficient signal-to-noise ratio for ERP analysis. The P300 component was quantified as the mean amplitude within the 300–500 ms time window. ERP analyses focused on frontal (F3, F4, Fz), central (C3, C4, Cz), parietal (P3, P4, Pz), and occipital (O1, O2, Oz) regions, consistent with previous semantic-related ERP studies (Hou & Lu, 2018). For each region, the mean amplitude and latency were calculated by averaging across the corresponding electrodes.

## 3 Results

### 3.1 Visual psychological evaluation of three kinds of flattened bamboo boards

#### 3.1.1 SD analysis

**Figure 4:**
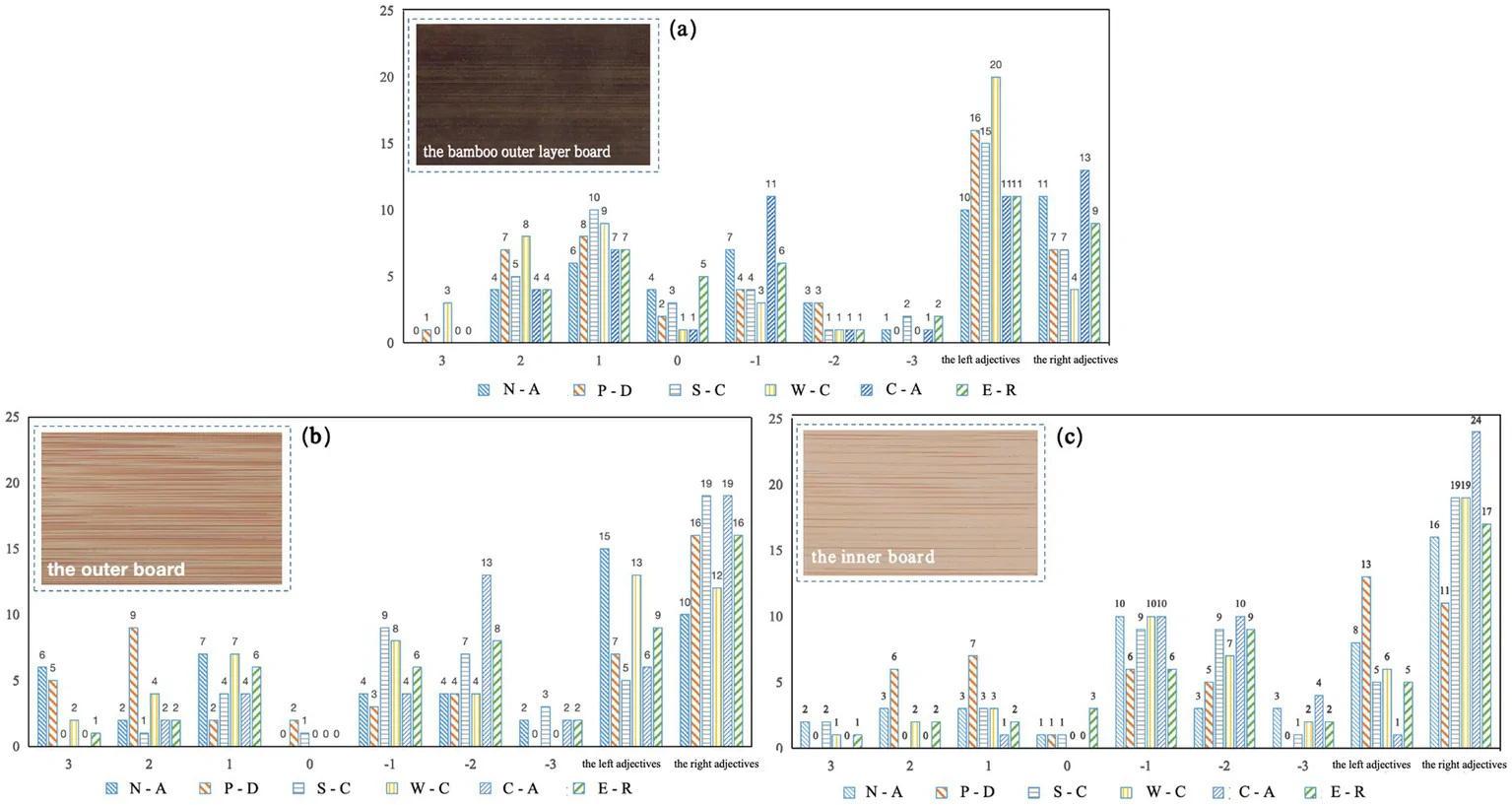
Visual psychological investigation results of three kinds of flattened bamboo boards based on the SD method. **(a)** The bamboo outer layer board; **(b)** the outer board; **(c)** the inner board. N-A, Natural–Artificial; P-D, Practical–Decorative; S-C, Smooth–Coarse; W-C, Warm–Cool; C-A, Comfortable–Afflictive; E-R, Exquisite–Rough.

Figure 4 presents the frequency distribution of participants’ subjective visual evaluations of the bamboo outer layer board, outer board, and inner board, whereas the corresponding mean scores, standard deviations, and statistical comparisons are summarized in Table 2. Overall, participants’ ratings were primarily concentrated around scores of −2, −1, 1, and 2, indicating moderate perceptual intensity across most bipolar adjective dimensions.

**Table 2:** ANOVA analysis of visual psychological evaluation scores of the three kinds of flattened bamboo boards.

Bipolar adjectives	The score of the bamboo outer layer board (Mean ± Standard deviation)/μV	The score of the outer board (Mean ± Standard deviation) /μV	The score of the inner board (Mean ± Standard deviation)/μV	The values of *F*	The values of *p*
Natural–Artificial	−0.02 ± 1.27	0.33 ± 1.73	−0.38 ± 1.54	1.399	0.253
Practical–Decorative	0.41 ± 1.16	0.76 ± 1.48	0.02 ± 1.24	2.374	0.101
Smooth–Coarse	0.18 ± 1.10	−0.82 ± 1.13	−0.66 ± 1.17	5.544	0.006**
Warm–Cool	0.96 ± 1.10	0.20 ± 1.25	−0.61 ± 1.24	10.264	0.000**
Comfortabl–Afflictive	−0.06 ± 1.16	−0.87 ± 1.30	−1.23 ± 0.75	7.823	0.001**
Exquisite–Rough	0.02 ± 1.10	−0.43 ± 1.36	−0.73 ± 1.23	2.562	0.084

**p < 0.01.

For the bamboo outer layer board (Figure 4a and Table 2), the mean scores for the Natural–Artificial (−0.02) and Exquisite–Rough (0.02) dimensions were close to zero, indicating relatively balanced perceptions of naturalness versus artificiality and delicacy versus roughness. In contrast, the board received the highest positive score in the Warm–Cool dimension (0.96), suggesting a stronger perception of coolness. The frequency distribution further revealed considerable variability in participants’ evaluations, particularly in the comfort-related dimensions, reflecting substantial individual differences in visual perception.

As shown in Figure 4b and Table 2, the outer board tended to be perceived as more artificial (0.33) and decorative (0.76). Negative mean scores in the Smooth–Coarse (−0.82), Comfortable–Afflictive (−0.87), and Exquisite–Rough (−0.43) dimensions indicated that participants generally associated this material with smoothness, comfort, and delicacy.

In contrast, the inner board (Figure 4c and Table 2) exhibited the most positive perceptual profile. It obtained negative scores in the Natural–Artificial (−0.38), Warm–Cool (−0.61), Comfortable–Afflictive (−1.23), and Exquisite–Rough (−0.73) dimensions, indicating stronger associations with naturalness, warmth, comfort, and delicacy. These findings suggest that the inner board elicited the most favorable visual impressions among the three materials.

The repeated-measures ANOVA results revealed significant differences among the three board types in the Smooth–Coarse (*p* = 0.006), Warm–Cool (*p* < 0.001), and Comfortable–Afflictive (*p* = 0.001) dimensions. No significant differences were observed in the Natural–Artificial (*p* = 0.253), Practical–Decorative (*p* = 0.101), or Exquisite–Rough (*p* = 0.084) dimensions. These results indicate that participants primarily distinguished the three flattened bamboo boards based on perceived smoothness, thermal impression, and comfort, whereas perceptions of naturalness, practicality, and delicacy showed greater overlap across materials.

From a structural perspective, bamboo exhibits a higher density and smaller cell size compared to wood. Its primary components—fibers and parenchyma cells—are multi-layered, with thicker cell walls, making it inherently denser (Liese, 1998; Lian et al., 2020; An, 2016). As a result, the outer board and inner board were more frequently perceived as smooth and delicate. Previous studies have identified a significant positive correlation between texture density and warmth perception (Yu et al., 2004), which may explain why the inner board, characterized by a sparser surface texture and warm yellow color, evoked stronger warmth perceptions. Conversely, the outer board and bamboo outer layer board, which contain thicker-walled fiber sheaths, were more likely to elicit a cooler sensation among participants.

#### 3.1.2 ERP results

In the 300–500 ms time window, this study extracted the P300 amplitude (Table 2) and latency (Table 3) for the three types of flattened bamboo boards. Statistical analysis of the P300 amplitude (Table 3) revealed a significant difference in the parietal region among the three board types (*p* = 0.049). The outer board exhibited the highest P300 amplitude (2.88 μV), followed by the inner board (2.27 μV), while the bamboo outer layer board elicited the lowest amplitude (1.19 μV). Additionally, weak statistical differences were observed in the central (*p* = 0.076) and occipital (*p* = 0.06) regions, suggesting that while these regions also responded to the visual stimuli, the effects were less pronounced.

**Table 3:** ANOVA analysis of amplitude value of different brain regions of three kinds of flattened bamboo boards.

EEG component	Electrode positions	The fluctuation of the outer board (Mean ± Standard deviation)/μV	The fluctuation of the inner board (Mean ± Standard deviation)/μV	The fluctuation of the bamboo outer layer board (Mean ± Standard deviation)/μV	The values of *F*	The values of *p*
P300	Frontal region F	3.49 ± 0.62	3.63 ± 1.22	2.62 ± 1.56	0.628	0.565
	Central region C	4.22 ± 0.40	3.94 ± 0.87	2.67 ± 0.76	4.088	*0.076*
	Parietal region P	2.88 ± 0.52	2.27 ± 0.86	1.19 ± 0.51	5.226	0.049*
	Occipital region O	3.40 ± 0.16	2.90 ± 0.75	2.09 ± 0.51	4.677	*0.060*

Italic values indicate marginal statistical significance at *p* < 0.10.

As shown in Table 4, P300 latency exhibited highly significant differences across the central, parietal, and occipital regions, except for the frontal region. Among the three types of flattened bamboo boards, the outer board required the longest semantic evaluation time (460–470 ms), followed by the bamboo outer layer board (450–460 ms), while the inner board had the shortest evaluation time (440–450 ms).

**Table 4:** ANOVA analysis of the latency of electroencephalographic components in different brain regions for the three kinds of flattened bamboo boards.

EEG component	Electrode positions	The latency of the outer board (Mean ± Standard deviation)/ms	The latency period of the inner board (Mean ± Standard deviation)/ms	The latency of the bamboo outer layer board (Mean ± Standard deviation)/ms	The values of *F*	The values of *p*
P300	Frontal region F	467.33 ± 4.62	439 ± 6.43	453 ± 39.00	1.114	0.388
	Central region C	469.33 ± 6.43	441 ± 4.16	451. ± 1.15	31.489	0.001**
	Parietal region P	464.67 ± 7.57	446 ± 2.00	453 ± 1.15	12.702	0.007**
	Occipital region O	467.33 ± 4.62	447 ± 3.06	453 ± 1.15	31.792	0.001**

*p < 0.05, **p < 0.01.

**Figure 5:**
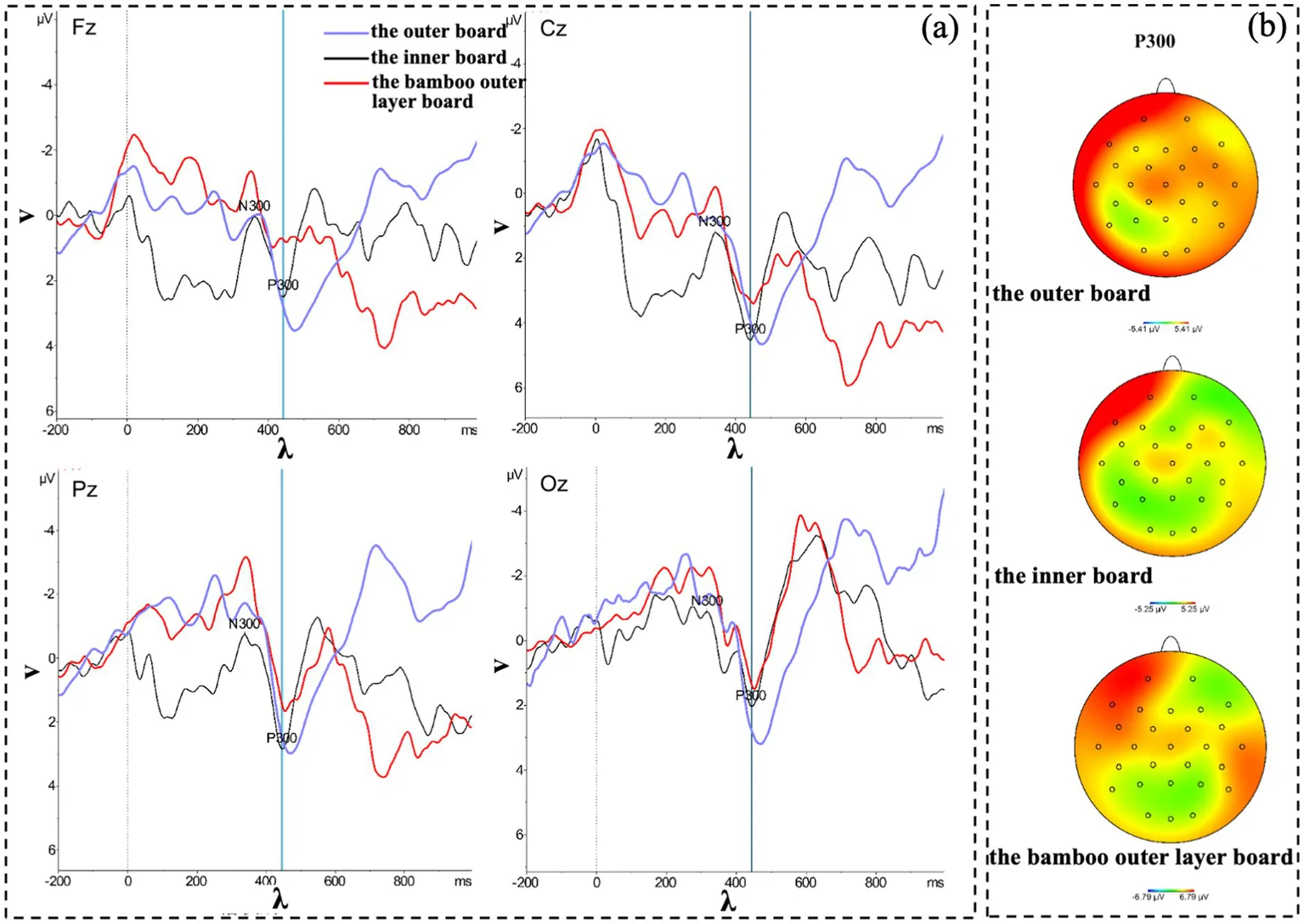
Average waveform diagrams and topographic maps for different flattened bamboo boards. **(a)** Waveforms of different electrode positions; **(b)** brain topographic map of three kinds of flattened bamboo boards.

Figure 5a illustrates the ERP waveforms recorded at different electrode positions. All three bamboo board types elicited a P300 positive potential within the 300–500 ms time window, confirming their impact on cognitive processing. Notably, analysis of P300 EEG waveforms from different brain regions revealed distinct differences, with the waveform of the bamboo outer layer board diverging significantly from those of the outer board and inner board, which exhibited similar waveform characteristics. The ERP topographical maps shown in Figure 5b suggest a relatively stronger distribution of neural activity in the left frontal region during the visual evaluation of flattened bamboo boards, while other brain regions also provided supportive contributions. This finding aligns with previous research emphasizing the frontal region’s role in executive judgment (Barbey et al., 2013). Moreover, varying levels of activation were detected across the scalp electrodes for all three types of bamboo boards, with significant differences observed particularly in the parietal region.

### 3.2 Psychological characteristics of subjects of different genders

**Figure 6:**
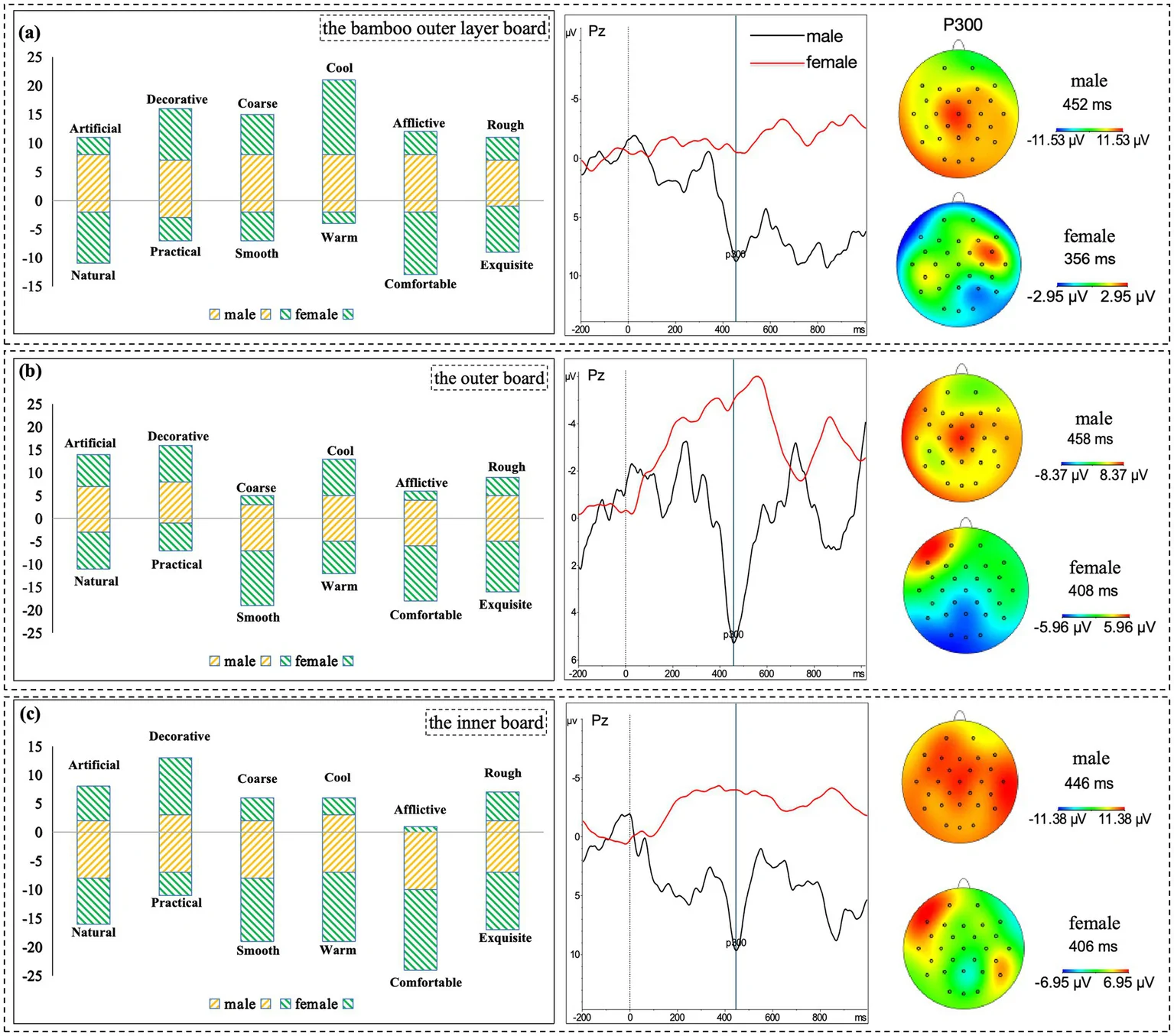
Subjective evaluation results, parietal waveforms, and P300 topographic map of three kinds of flattened bamboo boards by different genders. **(a)** Psychological evaluation of the bamboo outer layer board; **(b)** psychological evaluation of the outer board; **(c)** psychological evaluation of the inner board.

Visual psychological characteristics differ across gender and professional backgrounds (Barbey et al., 2013). To explore these differences in the perception of flattened bamboo boards, both subjective evaluations and event-related potential (P300 component) analyses were conducted, as illustrated in Figure 6 and Table 5.

**Table 5:** P300 amplitude value *t*-test of different brain regions between man and female under three kinds of flattened bamboo boards.

Specimen	Electrode positions	The amplitude value of males (Mean ± Standard deviation)/μV	The amplitude value of females (Mean ± Standard deviation)/μV	The values of *t*	The values of *p*
the outer board	Frontal region F	4.76 ± 0.48	0.36 ± 1.77	4.158	0.014*
	Central region C	6.21 ± 1.97	−2.27 ± 0.62	7.109	0.002**
	Parietal region P	4.31 ± 1.00	−3.61 ± 0.82	10.593	0.000**
	Occipital region O	4.47 ± 0.23	−3.89 ± 1.24	11.513	0.000**
the inner board	Frontal region F	9.64 ± 0.78	0.21 ± 1.13	13.744	0.000**
	Central region C	9.76 ± 1.53	−1.05 ± 0.25	12.056	0.000**
	Parietal region P	8.31 ± 1.16	−3.00 ± 0.74	14.238	0.000**
	Occipital region O	7.69 ± 0.13	−0.37 ± 1.42	9.787	0.010**
the bamboo outer layer board	Frontal region F	7.10 ± 2.26	0.64 ± 1.05	4.49	0.011*
	Central region C	8.88 ± 2.33	0.89 ± 0.30	5.899	0.026*
	Parietal region P	7.19 ± 1.64	−0.37 ± 1.33	6.207	0.003**
	Occipital region O	7.88 ± 0.31	−0.80 ± 0.66	20.761	0.000**

*p < 0.05, **p < 0.01.

The subjective evaluation results suggest that psychological responses between male and female participants varied primarily in the evaluative and emotional dimensions. In the assessment of the bamboo outer layer board, most males perceived it as having a rough surface and artificial appearance, which often evoked a sense of discomfort. In contrast, females found the same board to be more natural and delicate, associating it with a greater sense of comfort. A similar divergence was observed for the inner board, where males predominantly considered it practical, whereas females viewed it as decorative. The outer board, however, presented a more nuanced contrast; while most males perceived it as artificial, nearly half of the female participants described it as natural. These differences in perception may be attributed to the psychological stimuli induced by visual characteristics, such as the deep brown color of the bamboo outer layer board, which likely influenced emotional responses. Additionally, variations in personality traits between genders may have played a role, as males are often characterized as more outgoing and assertive, while females tend to be more gentle and detail-oriented in their cognitive evaluations.

Despite these differences, some commonalities in visual psychological perception were observed. Evaluations of the outer board remained consistent across five psychological dimensions for both genders, while the inner board showed alignment in five psychological measures and the bamboo outer layer board exhibited three shared psychological characteristics. These findings align with Liu Yixing’s research on the visual psychological perception of wood surfaces (Li et al., 1995), suggesting that certain fundamental perceptual attributes of flattened bamboo boards are not strongly influenced by gender differences.

Analysis of P300 component amplitude values for visual brainwave responses (Table 5) further confirmed significant gender-based differences (*p* < 0.05) across all three types of bamboo boards, with females exhibiting consistently lower amplitudes than males. The latency of semantic evaluations also differed significantly, with males requiring longer processing times compared to females. Specifically, for the outer board, male participants required 458 ms, while females completed the evaluation in 408 ms. Similarly, for the bamboo outer layer board, males averaged 452 ms, whereas females responded significantly faster at 356 ms. A comparable trend was noted for the inner board, where males required 446 ms, while females responded within 406 ms. These results suggest that males generally required more cognitive resources for decision-making, leading to longer evaluation times, whereas females demonstrated a more intuitive and rapid assessment process.

The ERP waveforms recorded during the 300–500 ms time window (Figure 6) further supported these findings, revealing that male participants exhibited larger P300 amplitudes, whereas female participants displayed lower amplitudes. The ERP topographical maps illustrated distinct scalp electrode activation patterns between genders. In males, P300 activations were primarily concentrated in the central, parietal, and occipital regions, while in females, activations were predominantly localized in the frontal region, except in the case of the bamboo outer layer board, where a more distributed activation pattern was observed. These activation patterns suggest that males engage in deeper cognitive processing when evaluating material surfaces, while females rely more on frontal-lobe-mediated executive functions and intuitive judgments.

## 4 Discussion

Event-related potentials (ERPs) reflect changes in brain electrical activity that occur in a time-locked relationship with stimuli, providing insight into neurophysiological mechanisms underlying cognitive processing (Mumtaz et al., 2018). Given their high temporal resolution, ERPs offer an objective measure of psychological activity and can effectively capture cognitive and emotional responses to visual stimuli (Kamarajan & Porjesz, 2015). This study primarily focused on the P300 component, a late positive potential associated with decision-making execution, inhibitory control processing, and cognitive resource allocation (Deng et al., 2021). The amplitude of the P300 reflects the degree of cortical arousal and the allocation of attentional resources, while its latency is indicative of the time required for stimulus evaluation and response selection (Hamidovic & Wang, 2019; Wang et al., 2012).

The results of this study revealed that the outer board elicited the highest P300 amplitude and the longest latency, suggesting that participants allocated the greatest cognitive resources and required longer processing times when evaluating its visual characteristics. Although participants tended to associate the outer board with greater artificiality and decoration than the inner board, these differences were not statistically significant. In contrast, significant differences were observed in the smooth–coarse, warm–cool, and comfortable–afflictive dimensions. The outer board was perceived as relatively cooler and less comfortable and elicited the highest P300 amplitude and longest latency, indicating greater cognitive effort and evaluative complexity during visual processing. This effect is likely due to the visually rich surface elements of the dark red outer board, which include material color and distinct textures, potentially interfering with participants’ visual psychological judgments when compared to the bright yellow inner board and the dark brown bamboo outer layer board. Notably, the inner board, with its bright yellow hue, may have exerted a cognitive priming effect, enhancing attentional focus and facilitating more rapid emotional judgments (Luo et al., 2020).

Conversely, the bamboo outer layer board elicited the lowest P300 amplitude but had the second-longest latency, suggesting a relatively lower cognitive load but a more prolonged decision-making process. This may be attributed to the dual nature of its visual attributes, as it exhibits both natural and artificial elements, appearing decorative yet rough, cool yet comfortable. Such contrasting perceptions might have resulted in a more nuanced and complex evaluative process, where participants required additional time to reconcile conflicting visual cues (Lian et al., 2022). The relatively low amplitude values for the bamboo outer layer board align with previous findings indicating that pleasant images tend to evoke smaller P300 amplitudes and longer latencies than unpleasant stimuli (Campanella et al., 2004; Macnamara et al., 2009). This suggests that while the inner board was perceived as inviting and warm, the bamboo outer layer board was associated with aesthetic neutrality and subtle comfort, particularly in warm environments, making it more psychologically engaging but cognitively less demanding.

Previous research has established that gender differences influence cognitive and emotional processing, with variations in neural activation patterns, decision-making strategies, and affective responses (Cahill, 2016; Nanova et al., 2022). The present study confirmed that female participants exhibited consistently lower P300 amplitudes and shorter latencies than their male counterparts across all three board types. Given that latency represents the ease of neural processing, these findings suggest that females required fewer cognitive resources to assess the visual attributes of the bamboo boards, demonstrating more intuitive and rapid evaluations (Feng et al., 2016; Sachs et al., 2004).

Neurophysiological analyses further revealed that while males exhibited widespread neural activation across the central, parietal, and occipital regions, females primarily engaged the frontal region, except when evaluating the bamboo outer layer board. This suggests that males relied more on visual–spatial processing and detailed cognitive assessment, whereas females processed the stimuli in a more holistic and affect-driven manner, which aligns with established research on gender-specific neural pathways in aesthetic and emotional evaluations. The subjective psychological evaluations of the boards further reinforced these differences. Males tended to perceive the outer board as artificial, the inner board as practical, and the bamboo outer layer board as artificial and rough, whereas females described the outer board as more natural, the inner board as decorative, and the bamboo outer layer board as natural and delicate. These distinctions underscore how gender-related perceptual biases influence material esthetics and comfort assessments (Cipolli, 2021; Meyers-Levy & Zhu, 2010).

Additionally, the ERP amplitude values for positive emotions were consistently higher in males than females, reflecting greater neural engagement in affective processing. Despite these differences, both male and female participants exhibited an overall preference for the inner board, as indicated by its highest ERP amplitude in males (9.76 μV) and the lowest in females (0.21 μV). This reinforces the notion that regardless of gender, the inner board was perceived as the most esthetically and emotionally appealing option, likely due to its warm coloration and smooth texture.

The findings of this study have important implications for interior material design, product esthetics, and consumer preferences. The strong cognitive and affective responses elicited by the outer board suggest that its deep red coloration and pronounced textures may require careful consideration in interior applications, as they could evoke greater psychological engagement and potentially cognitive fatigue over time (Zhang & Han, 2013). The deeper red coloration of the outer board results from its high vascular bundle density, which lowers porosity and enhances light absorption, giving rise to a visually heavier and cooler appearance (Lian et al., 2022). Thermal softening of this dense microstructure facilitates chromophore formation, further strengthening the visual impact. Consequently, the outer board may be more suitable for interior environments that favor cooler tones, strong visual contrast, or spaces requiring a more structured and less absorptive surface.

In contrast, the inner board’s bright yellow hue and smooth surface provided a more inviting and comforting experience, making it a suitable choice for residential and relaxation spaces. Compared with the outer layer, the inner layer contains a higher proportion of parenchyma cells and exhibits greater porosity, contributing to its warmer visual tone and softer texture (Liese, 1998; Lian et al., 2022). The large, thin-walled parenchyma cells promote diffuse light scattering, producing a visually softer and more homogeneous surface that facilitates perceptual fluency and enhances perceived comfort (Chen et al., 2023). These characteristics not only enhance psychological warmth but also imply potential advantages in acoustic absorption or sound insulation, making the inner board an attractive option for living rooms, bedrooms, reading spaces, or interiors adopting warm color palettes.

Meanwhile, the bamboo outer layer board’s neutral yet distinctive aesthetic allows it to function as a versatile material in interiors requiring a balance between natural expression and visual restraint. Although the bamboo outer layer board displays the darkest coloration among the three types, this effect is mainly attributable to the waxy epidermal layer (Chen et al., 2023; Jiang, 2002). The wax layer increases surface gloss and smoothness and contributes to a more uniform fiber configuration, producing a visually refined, lustrous surface with moderated chromatic intensity compared with the outer board (Chen et al., 2023). These characteristics make the bamboo outer layer board particularly suitable for interior environments that favor cool color schemes, vintage or retro-inspired styles, or spaces that seek a visually rich yet subtly refined material presence.

Several limitations of this study should be acknowledged. First, the Semantic Differential (SD) evaluation was conducted as part of the ERP experimental procedure and involved the same participant group (n = 25). Therefore, the SD findings were primarily intended to provide perceptual context for interpreting the ERP results rather than to establish broadly generalizable perceptual characteristics. Second, although the sample size is comparable to that commonly adopted in ERP studies, it may limit the generalizability of the SD findings and gender-related analyses. Consequently, these results should be regarded as preliminary and warrant further validation using larger and more diverse participant populations.

Overall, integrating SD-based psychological evaluations with ERP measurements and anatomical–chemical evidence provides a coherent mechanistic explanation for how bamboo surface microstructure relates to affective appraisal, perceptual fluency, and cognitive processing. Vascular bundle density, parenchyma-cell distribution, wax-layer composition, chromophore formation, and optical scattering characteristics collectively determine how bamboo materials are visually perceived and psychologically evaluated. These findings underscore the relevance of considering both sensory psychology and material science when examining how bamboo surfaces are visually perceived and cognitively evaluated. By bridging microstructural attributes with neural and affective responses, this study advances interdisciplinary understanding and offers evidence-based guidance for creating visually harmonious, cognitively comfortable, and emotionally supportive interior environments using bamboo materials.

## 5 Conclusion

This study investigated the visual psychological characteristics of three types of flattened bamboo boards by integrating semantic differential (SD) evaluations with event-related potential (ERP) measurements. The results demonstrated that variations in surface appearance among the three boards elicited distinct affective and cognitive responses, reflected in both subjective evaluations and neural indicators. Differences in perceived naturalness, warmth, comfort, and texture highlighted the important role of visual attributes in shaping material perception.

Among the three board types, the outer board was associated with stronger visual stimulation and greater evaluative difficulty, as evidenced by its highest P300 amplitude and longest latency, indicating increased cognitive resource allocation and greater evaluative complexity. In contrast, the inner board, characterized by its bright yellow hue and relatively smooth appearance, received the most positive perceptual evaluations, particularly in the warm–cool and comfortable–afflictive dimensions, and was processed with the lowest cognitive load, suggesting greater perceptual fluency and ease of evaluation. The bamboo outer layer board evoked a more heterogeneous perceptual profile, balancing natural and artificial qualities as well as exquisiteness and roughness. It was more frequently associated with coolness and required a longer evaluation time despite eliciting relatively lower cognitive effort.

ERP results further showed that cognitive processing of bamboo board visuals was primarily associated with frontal regions, while perceptual differences were reflected in central, parietal, and occipital regions, with the parietal area exhibiting the most pronounced activation. Gender-based analyses revealed that males generally allocated more cognitive resources and engaged broader neural networks during evaluation, whereas females demonstrated shorter processing times and relied more heavily on frontal-lobe activity, suggesting a more intuitive assessment style. Despite these differences, both male and female participants expressed positive emotional responses toward all three bamboo boards, with a shared preference for the inner board.

Overall, this study demonstrates that combining psychological evaluation methods with neurophysiological measures provides a robust framework for understanding how visual attributes of bamboo materials influence affective appraisal and cognitive processing. The findings offer practical insights for material selection and interior design, supporting the development of bamboo-based products and environments that are visually harmonious, cognitively comfortable, and emotionally supportive.

## Data Availability

The raw data supporting the conclusions of this article will be made available by the authors, without undue reservation.
